# Evidence of artemisinin partial resistance in northwestern Tanzania: clinical and molecular markers of resistance

**DOI:** 10.1016/S1473-3099(24)00362-1

**Published:** 2024-11

**Authors:** Deus S Ishengoma, Celine I Mandara, Catherine Bakari, Abebe A Fola, Rashid A Madebe, Misago D Seth, Filbert Francis, Creyton C Buguzi, Ramadhan Moshi, Issa Garimo, Samwel Lazaro, Abdallah Lusasi, Sijenunu Aaron, Frank Chacky, Ally Mohamed, Ritha J A Njau, Jovin Kitau, Charlotte Rasmussen, Jeffrey A Bailey, Jonathan J Juliano, Marian Warsame

**Affiliations:** a National Institute for Medical Research, Dar es Salaam, Tanzania; b Department of Biochemistry, Kampala International University in Tanzania, Dar es Salaam, Tanzania; c Department of Pathology and Laboratory Medicine, Brown University, Providence, RI, USA; d Centre for Computational Molecular Biology, Brown University, Providence, RI, USA; e National Malaria Control Program, Dodoma, Tanzania; f Department of Parasitology and Medical Entomology, School of Public Health and Social Sciences, Muhimbili University of Health and Allied Sciences, Dar es Salaam, Tanzania; g World Health Organization Country Office, Dar es Salaam, Tanzania; h World Health Organization, Geneva, Switzerland; i Division of Infectious Diseases, University of North Carolina School of Medicine, University of North Carolina at Chapel Hill, Chapel Hill, NC, USA; j Department of Public Health and Community Medicine, Gothenburg University, Gothenburg, Sweden; k Research Unit in Rector's Office, Benadir University, Mogadishu, Somalia

## Abstract

**Background:**

In 2021, nationwide malaria molecular surveillance revealed a high prevalence of a validated artemisinin resistance marker, the *kelch13* (*k13*) Arg561His mutation, in the Kagera region of northwestern Tanzania. We aimed to investigate the efficacy of artemether–lumefantrine and artesunate–amodiaquine and to confirm the presence of artemisinin partial resistance (ART-R) in the Karagwe district of this region.

**Methods:**

This single-arm, therapeutic efficacy study was carried out at the Bukangara dispensary in the Karagwe district of the Kagera region in northwestern Tanzania. Eligible participants were aged between 6 months and 120 months, had confirmed *Plasmodium falciparum* asexual parasitaemia, and met other inclusion criteria according to WHO's standard protocol. Participants were enrolled, treated sequentially with either artemether–lumefantrine or artesunate–amodiaquine, and assessed clinically and parasitologically for 28 days as per WHO protocol. Parasitaemia was measured every 8 h until day 3, on day 7, and then during weekly follow-up visits until day 28. Mutations in the *k13* gene and extended haplotypes with the mutations were analysed, and comparisons were made with previous samples collected in the same region of Kagera and in Rwanda and southeast Asia. The primary endpoint was PCR-corrected cure rate.

**Findings:**

Between April 29 and Sept 1, 2022, 343 patients were screened, of whom 176 were enrolled: 88 in each treatment group. The PCR-corrected cure rate was 98% (95% CI 91–100) in the artemether–lumefantrine group and 100% (96–100) in the artesunate–amodiaquine group. Persistent parasitaemia on day 3 occurred in 11 (13%) of 88 patients in the artemether–lumefantrine group and 17 (19%) of 88 patients in the artesunate–amodiaquine group. Arg561His mutations on day 0 and parasitaemia on day 3 were reported in eight (9%) of 87 patients in the artemether–lumefantrine group and ten (12%) of 86 patients in the artesunate–amodiaquine group. The median parasite clearance half-life in patients harbouring parasites with Arg561His mutation was 6·1 h in the artemether–lumefantrine group and 6·0 h in the artesunate–amodiaquine group. Parasites with the Arg561His mutation were not similar to those from southeast Asia and Rwanda but had similar haplotypes to parasites reported in the same Tanzanian region of Kagera in 2021.

**Interpretation:**

This study confirms the presence of ART-R in Tanzania, although artemether–lumefantrine and artesunate–amodiaquine showed high efficacy. A context-specific response strategy and vigilance to detect the reduced efficacy of current antimalarial treatments and ART-R in other parts of the country are urgently needed.

**Funding:**

The Bill & Melinda Gates Foundation and the US National Institutes of Health.

## Introduction

Artemisinin-based combination therapies (ACTs) are recommended for the treatment of uncomplicated malaria caused by *Plasmodium falciparum*. These ACTs include artemether–lumefantrine, artesunate–amodiaquine, dihydroartemisinin–piperaquine, artesunate–mefloquine, artesunate–sulfadoxine–pyrimethamine, and artesunate–pyronaridine. WHO recommends monitoring the efficacy of recommended ACTs through therapeutic efficacy studies every 2 years, using the standard protocol to inform treatment policy.^1^ High efficacy (>90%) of artesunate–amodiaquine and artemether–lumefantrine has been reported in most countries in Africa, although efficacies of artemether–lumefantrine below the 90% threshold recommended for treatment policy change have also been reported in Angola, Burkina Faso, the Democratic Republic of the Congo, and Uganda.^1^, ^2^, ^3^, ^4^, ^5^ However, these studies deviated from the WHO-recommended PCR correction on which the 90% cutoff was based.^6^

Artemisinin partial resistance (ART-R) is confirmed in an area when more than 5% of patients treated with an ACT or artesunate monotherapy harbour parasites with mutations validated to be associated with resistance and have delayed parasite clearance, described as either persistent parasitaemia at day 3 or a parasite clearance half-life (PCT_1/2_) of 5 h or more.^7^ To date, 13 *kelch13* (*k13*) mutations have been validated as associated with ART-R (Phe446Ile, Asn458Tyr, Cys469Tyr, Met476Ile, Tyr493His, Arg539Thr, Ile543Thr, Pro553Leu, Arg561His, Pro574Leu, Cys580Tyr, Arg622Ile, and Ala675Val) and nine *k13* mutations are considered candidate or associated markers (Pro441Leu, Gly449Ala, Cys469Phe, Ala481Val, Arg515Lys, Pro527His, Asn537Ile [or Asn537Asp], Gly538Val, and Val568Gly).^8^ ART-R was reported in southeast Asia in 2008 and has more recently been confirmed in Africa—in Rwanda (2018), Uganda (2019), and Eritrea (2019).^9^, ^10^, ^11^, ^12^, ^13^

**Research in context**

**Evidence before this study**

Artemisinin partial resistance (ART-R) is defined as delayed clearance of a parasite strain carrying a validated marker of artemisinin resistance after treatment with an artemisinin-based combination therapy (ACT) or artesunate monotherapy. At present, 13 different *kelch13* (*k13*) mutations have been validated as markers of artemisinin resistance. ART-R is confirmed in an area if a quality-controlled study using an ACT or artesunate monotherapy finds that more than 5% of patients have parasites with validated *k13* mutations and delayed clearance (either persistent parasitaemia detected by microscopy on day 3 or a parasite clearance half-life of ≥5 h). ART-R was first reported in Cambodia in 2008 and later in several countries in southeast Asia. We searched PubMed for articles published between Jan 1, 2014, and Dec 31, 2023, using the terms “artemisinin”, “artemisinin partial resistance”, “artemisinin-based combination therapies”, “kelch 13”, and “therapeutic efficacy studies” in combination with “Africa” or “Tanzania”. The 13 publications retrieved confirmed the emergence of ART-R associated with *k13* mutations resulting in the following amino acid changes: Arg561His in Rwanda, Ala675Val and Cys469Tyr in Uganda, and Arg622Ile in Eritrea. In all these studies, the tested ACTs resulted in a high cure rate. The Arg622Ile mutant has not been reported in southeast Asia but is circulating in Eritrea, Ethiopia, Sudan, and Somalia within the Horn of Africa. In Tanzania, a nationwide malaria molecular surveillance, launched in January, 2021, showed a high prevalence of the Arg561His mutation in the northwestern region of Kagera, close to the border with Rwanda and Uganda.

**Added value of this study**

This therapeutic efficacy study provides the first evidence, to our knowledge, of ART-R in the Kagera region of northwestern Tanzania, an area close to the border with Rwanda and Uganda, where ART-R has been previously confirmed. Tanzania is therefore the fourth country in Africa in which ART-R has been confirmed. These findings suggest that ART-R is rapidly evolving and could be found in more areas of Africa. Parasites with the Arg561His mutation were not similar to those from southeast Asia and Rwanda, but had similar haplotypes to parasites reported in the Kagera region of Tanzania in 2021. Furthermore, this study shows that both artemether–lumefantrine and artesunate–amodiaquine remain highly efficacious for the treatment of uncomplicated falciparum malaria among patients in the study area, as previously reported in Rwanda, Uganda, and Eritrea.

**Implications of all the available evidence**

The emergence of confirmed ART-R in Africa, so far in four countries (Eritrea, Rwanda, Tanzania, and Uganda), poses a serious threat to malaria control in Africa, which accounts for more than 95% of the global malaria burden. The current evidence for ART-R in the Kagera region of Tanzania calls for an urgent response, including the development of a context-specific strategy based on the WHO strategy launched in 2022, to respond to antimalarial drug resistance in Africa. The fact that ART-R has been confirmed in the Kagera region—an area with considerable human movement near to the borders with Rwanda and Uganda, where resistance has also been reported—calls for cross-border collaboration to harmonise strategies to combat this threat in the Great Lakes region of Africa. Nationwide studies of molecular markers in Tanzania, which revealed a high prevalence of validated *k13* mutations in the Kagera region, informed the location of the current study. Malaria molecular surveillance, including surveillance of markers of drug resistance to different antimalarials, could therefore have an important role in informing targeted therapeutic efficacy studies and confirming ART-R in other parts of Tanzania and in other countries.

In Tanzania, artemether–lumefantrine is recommended as first-line treatment of uncomplicated falciparum malaria, with artesunate–amodiaquine recommended as an alternative ACT.^14^ Routine therapeutic efficacy studies of both combination therapies have shown high efficacy to date.^15^, ^16^ However, the 2021 nationwide malaria molecular surveillance found a hotspot with a high prevalence of the *k13* Arg561His mutation in the Karagwe district of the Kagera region of Tanzania, as reported in a preprint paper.^17^ These findings prompted this study, which aimed to assess the efficacy of artemether–lumefantrine and artesunate–amodiaquine and confirm the presence of ART-R in the Karagwe district, where there is considerable population movement across the borders with Rwanda and Uganda.^18^

## Methods

### Study design and participants

**Figure 1:**
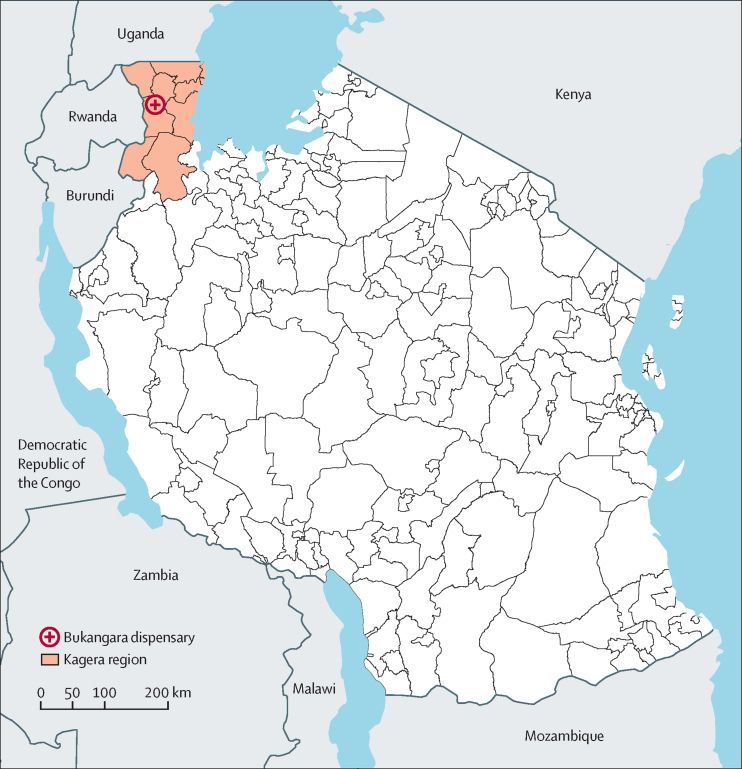
Map showing the location of Bukangara dispensary in the Karagwe district of the Kagera region, northwestern Tanzania

This single-arm therapeutic efficacy study was carried out between April 29 and Sept, 1, 2022, at the Bukangara dispensary in the Karagwe district of the Kagera region of Tanzania (figure 1).^17^ The site is about 100 km from Rukara, Rwanda, where ART-R was confirmed in 2018.^11^

Screening for eligibility, enrolment, treatment, and follow-up was done by trained clinical officers according to the WHO protocol for therapeutic efficacy studies (appendix pp 2–3).^19^ Targeted patients were aged between 6 months and 120 months; had fever at presentation (defined as an axillary temperature of at least 37·5°C), a history of fever in the previous 24 h, or both; had *P falciparum* parasitaemia of 500–200 000 asexual parasites per μL of blood by microscopy; had a haemoglobin concentration of at least 8 g/dL; and were able and willing to attend scheduled follow-up visits. Other inclusion and exclusion criteria were according to the WHO protocol (appendix p 3).^19^ Enrolment was done sequentially, starting with the artemether–lumefantrine group until the target sample size was reached, after which enrolment for the artesunate–amodiaquine group was initiated. Infected patients who were not eligible for enrolment in the study were treated according to national treatment guidelines.^14^ More details of the methods are provided in the appendix (pp 2–5).

Ethical clearance was obtained from the Medical Research Coordinating Committee (MRCC) of the Tanzanian National Institute for Medical Research (NIMR; NIMR/HQ/R.8c/Vol.I/976), and the WHO Ethics Review Committee (Tanzania Kagera 2021). Additional permission was obtained from the President's Office, Regional Administration and Local Government. Parents or guardians of study children were provided with details of the study and gave written informed consent before recruitment. Free health care was provided to all enrolled patients throughout the follow-up period. Parents and guardians were provided with US$2 (about 5400 Tanzanian shillings) as travel expenses per visit. The principal investigator ensured that all screening forms, case report forms, and the completed subject identification code list (patient screening and enrolment logbooks) were kept in locked files that only study personnel could access.

### Procedures

#### Treatment and follow-up procedures

In the artemether–lumefantrine group, patients received an oral twice-daily dose ranging between 5 mg and 24 mg of artemether and 29 mg and 144 mg of lumefantrine per kg bodyweight (without food) for 3 days according to the manufacturer's instructions (Cipla; Mumbai, India). Patients in the artesunate–amodiaquine group received an oral daily dose ranging between 2 mg and 10 mg of artesunate and 7·5 mg and 15 mg of amodiaquine per kg bodyweight for 3 days according to the manufacturer's instructions (Winthrop, Sanofi Aventis; Casablanca, Morocco). The study drugs were provided by WHO. All doses were administered under the direct observation of an experienced nurse and a repeat dose was given if vomiting occurred within 30 min. Patients with persistent vomiting were excluded from the study and treatment was administered parenterally according to national treatment guidelines.^14^ Enrolled patients were retained at the study facility for 3 days, during which they had clinical assessments and parasitological assessments at 8-h intervals to measure the PCT_1/2_. Clinical and parasitological assessments were also conducted during scheduled follow-up visits on days 7, 14, 21, and 28, or during unscheduled visits at any time if the child felt unwell. Patients who missed their scheduled visits were visited at home for clinical and parasitological assessments. Patients with parasite recurrence were given artemether–lumefantrine if they were in the artesunate–amodiaquine group or artesunate–amodiaquine if they were in the artemether–lumefantrine group. At each follow-up visit, the occurrence and nature of adverse events and serious adverse events were assessed using a standard case report form, defined and managed according to the standard WHO protocol.^19^ Any serious adverse event was to be reported to the NIMR, WHO, and the NIMR-MRCC.

#### Microscopy examination and haemoglobin blood concentration

Thick and thin blood smears were collected, stained with Giemsa, and examined for the presence of *P falciparum* and to estimate parasite density before treatment (day 0) and at each scheduled (days 1, 2, 3, 7, 14, 21, and 28) or unscheduled assessment according to WHO protocol.^19^ To estimate PCT_1/2_, blood smears were taken every 8 h until two consecutive smears were negative or the patient reached day 3 without clearing the parasites. Haemoglobin concentrations (g/dL) were measured on day 0 with a HemoCue machine (HemoCue; Ångelholm, Sweden) to identify patients with anaemia (<8 g/dL), who were excluded from the study.

#### Parasite genotyping

Blood samples were collected as dried blood spots on Whatman grade 3 filter papers (GE Healthcare Life Sciences; Chicago, IL, USA), on day 0 and on the day of parasite recurrence (from day 7 onwards), and used for genotyping of malaria parasites. DNA was extracted from dried blood spots using a QIAamp DNA Blood Midi Kit (Qiagen; Hilden, Germany). Paired samples (pre-treatment and during recurrence) were genotyped for genes encoding merozoite surface proteins 1 and 2 (*msp1* and *msp2*) and glutamate rich protein (*glurp*). Gel analyser version 19.1 was used to estimate fragment sizes and the bins used to define a match were 10 bp for *msp1* and *msp2*, and 50 bp for *glurp*. The WHO decision algorithm was used to classify recrudescence and new infection.^20^

#### Molecular markers of antimalarial drug resistance

DNA extracted from pre-treatment and recurrent samples was analysed for mutations in the *k13* gene (codons 430–720), which is linked to ART-R,^21^ and the *P falciparum* multidrug resistance 1 gene (*Pfmdr1*; codons 86, 184, and 1246), which is suspected to be associated with reduced sensitivity to 4-aminoquinolines, using capillary sequencing according to published protocols.^16^ SNP calls in *Pfmdr1* and *k13* were done using Geneious analysis software (version 2022.2.2) by mapping the sequence data on the 3D7 reference sequences.^16^

Whole-genome sequencing was done on 83 DNA samples (49 with *k13* Arg561His mutations and 34 wild type) to assess extended haplotypes around the gene. Selective whole-genome amplification and sequencing were done as previously described.^22^ For comparison, whole-genome sequencing data of Arg561His mutants were downloaded from public databases and included samples from Rwanda (n=25),^23^ the 2021 survey of the Kagera region (n=9),^17^ and southeast Asia (n=42).^24^ The data were processed accordingly and pairwise comparisons were made across the four datasets (appendix pp 6–7).

### Outcomes

Treatment outcomes were classified as early treatment failure, late clinical failure, late parasitological failure, or adequate clinical and parasitological response (ACPR), according to the WHO protocol.^19^ The primary endpoint was the PCR-adjusted ACPR at the day-28 follow-up. Secondary endpoints were persistent parasitaemia on day 3, PCT_1/2_, polymorphism in *k13* and *Pfmdr1*, and the occurrence of adverse and serious adverse events.

### Statistical analysis

We assumed that 5% of patients treated with either artemether–lumefantrine or artesunate–amodiaquine were likely to have treatment failure. With a confidence level of 95%, an estimated 5% margin of error, and 20% loss to follow-up or withdrawals, the sample size was 88 patients per drug, as per WHO protocol.^19^

Data were double-entered and analysed using STATA for Windows, version 17 and WHO Excel software. Treatment outcomes were analysed using both per-protocol and Kaplan–Meier methods. Patients who were lost to follow-up or withdrew during follow-up or who had reinfection or indeterminate PCR results were excluded from the PCR-corrected per-protocol analysis. For the Kaplan–Meier analysis, patients who were lost to follow-up or withdrew during follow-up were censored on the last day of follow-up, those with a new infection were censored on the day of reinfection, and those with indeterminate PCR results were excluded. The PCT_1/2_ was calculated using the parasite clearance estimator and a value of 5∤0 h or more was considered to indicate delayed parasite clearance, as previously described.^25^ Descriptive statistics, including proportions, percentages, means with SD, and medians with IQR, were reported as appropriate. χ^2^ or Fisher's exact tests were used to compare categorical data. The normality of distribution for continuous variables was assessed using histograms, Q–Q plots, and the Shapiro–Wilk test. The distribution of sample variance in each group was assessed using Lavene's test for equality of variances. Continuous variables were compared using *t* tests (for normally distributed data) or the Mann–Whitney *U* test (a non-parametric test for non-normally distributed data). Wilson-score Agresti method was used to calculate 95% CIs for binomial proportions. Two-sided p values of 0·05 or less were considered statistically significant.

### Role of the funding source

The funders of the study had no role in study design, data collection, data analysis, data interpretation, or writing of the report.

## Results

Between April 29 and Sept 1, 2022, we screened 343 patients, 176 (51%) of whom were recruited: 88 to the artemether–lumefantrine group and 88 to the artesunate–amodiaquine group. All patients completed their follow-up visits without loss to follow-up or withdrawal (appendix p 15). Baseline characteristics (age, anthropometric measurements, haemoglobin concentrations, axillary temperature, and parasitaemia) were similar in both groups (p>0·05; table 1).

**Table 1:** Baseline characteristics

		**Artemether–lumefantrine (n=88)**	**Artesunate–amodiaquine (n=88)**	**p value**
Median age, years (IQR)		4·0 (2·0–6·0)	4·0 (3·0–8·0)	0·17
Age group				
	<5 years	57 (65%)	49 (56%)	0·22
	5–10 years	31 (35%)	39 (44%)	..
Weight, kg		14·2 (4·6)	14·8 (4·9)	0·42
Upper mid-arm circumference, mm		152·7 (15·5)	150·0 (12·0)	0·50
BMI, kg/m^2^		14·0 (1·3)	14·3 (1·3)	0·87
Haemoglobin concentration, g/dL		10·8 (1·3)	10·8 (1·6)	0·97
Axillary temperature, °C		37·9 (1·2)	37·7 (1·3)	0·35
Parasitaemia, GMPD per μL (95% CI)		19 448 (14 630–25 854)	17 811 (13 164–24 097)	0·80

Data are n (%) or mean (SD) unless otherwise stated. GMPD=geometric mean parasite density.

31 (35%) of 88 patients treated with artemether–lumefantrine had recurrent infections and the PCR-uncorrected ACPR was 65% (95% CI 54–75; table 2). These recurrent infections occurred in three (10%) of 31 patients on day 14, 17 (55%) on day 21, and 11 (35%) on day 28. In the artesunate–amodiaquine group, two (2%) of 88 patients had a recurrence of infection, one (50%) on day 14 and one (50%) on day 21; the PCR-uncorrected ACPR in this group was 98% (92–100). The PCR-corrected ACPRs from the per-protocol analysis were 98% (91–100) for the artemether–lumefantrine group and 100% (96–100) for the artesunate–amodiaquine group. Kaplan–Meier PCR-corrected ACPRs were also 98% (91–100) for artemether–lumefantrine and 100% (96–100) for artesunate–amodiaquine (table 2). Kaplan–Meier survival curves for PCR-uncorrected and PCR-corrected outcomes are shown in the appendix (p 16). The reported adverse events are listed in the appendix (p 11); none were considered drug-related and there were no serious adverse events.

**Table 2:** PCR-uncorrected and PCR-corrected treatment outcomes

		**Artemether–lumefantrine (n=88)**	**Artesunate–amodiaquine (n=88)**
Parasitaemia on day 2		41 (47%, 37–57)	36 (41%; 31–51)
Parasitaemia on day 3		11 (13%, 7–21)	17 (19%; 12–29)
PCR-uncorrected			
	Early treatment failure	0 (0%; 0–4)	0 (0%; 0–4)
	Late clinical failure	5 (6%; 2–13)	1 (1%; 0–6)
	Late parasitological failure	26 (30%; 20–40)	1 (1%; 0–6)
	ACPR	57 (65%; 54–75)	86 (98%; 92–100)
	Total patients in per-protocol analysis	88	88
	Kaplan–Meier: cumulative cure rate	57 (65%; 54–74)	86 (98%; 91–100)
PCR-corrected			
	Early treatment failure	0 (0%; 0–6)	0 (0%; 0–4)
	Late clinical failure	0 (0%; 0–6)	0 (0%; 0–4)
	Late parasitological failure	1 (2%; 0–9)	0 (0%; 0–4)
	ACPR	57 (98%; 91–100)	86 (100%; 96–100)
	Total patients in per-protocol analysis	58	86
	New infections	28	2
	Not determined	2	0
	Kaplan–Meier: cumulative cure rate	57 (98%; 91–100)	86 (100%; 96–100)

Data are n (%; 95% CI) or n. ACPR=adequate clinical and parasitological response.

For both groups, both *k13* and region 1 of *Pfmdr1* were successfully sequenced—pre-treatment and on the recurrence of parasitaemia—in more than 94% of samples. Sequencing success was lower for region 2 of *Pfmdr1*, ranging from 42% to 92% in both groups (table 3). Arg561His mutations were detected in 21 (24%) of 87 pre-treatment samples in the artemether–lumefantrine group and 18 (21%) of 86 pre-treatment samples in the artesunate–amodiaquine group. In region 1 of the *Pfmdr1* gene, all successfully analysed samples carried the Asn86 wild type, and the Tyr184Phe mutation was detected in 37 (42%) of 88 pre-treatment samples in both groups. In *Pfmdr1* region 2, four samples (three [4%] of 81 pre-treatment samples and one [3%] of 29 samples from recurrent parasitaemia) from the artemether–lumefantrine group had the Asp1246Tyr mutation (table 3). Details of the association between mutations in the *Pfmdr1* gene and treatment outcomes are provided in the appendix (pp 7–8).

**Table 3:** Mutations in *k13* and *Pfmdr1*

	**Artemether–lumefantrine**		**Artesunate–amodiaquine**	
	Day 0	Day of recurrent parasitaemia	Day 0	Day of recurrent parasitaemia
**Mutations in *k13***				
Successfully analysed	87/88 (99%)	29/31 (94%)	86/88 (98%)	2/2 (100%)
Wild type	66/87 (76%)	23/29 (79%)	68/86 (79%)	2/2 (100%)
Arg561His mutation	21/87 (24%)	6/29 (21%)	18/86 (21%)	0/2 (0%)
**Mutations in** Pfmdr1**; region 1**				
Successfully analysed	88/88 (100%)	29/31 (94%)	88/88 (100%)	2/2 (100%)
Asn86 wild type	88/88 (100%)	29/29 (100%)	88/88 (100%)	2/2 (100%)
Tyr184 wild type	51/88 (58%)	17/29 (59%)	51/88 (58%)	2/2 (100%)
Tyr184Phe mutation	37/88 (42%)	12/29 (41%)	37/88 (42%)	0/2 (0%)
**Mutations in** Pfmdr1**; region 2**				
Successfully analysed	81/88 (92%)	29/31 (94%)	42/88 (48%)	1/2 (50%)
Asp1246 wild type	78/81 (96%)	28/29 (97%)	42/42 (100%)	1/1 (100%)
Asp1246Tyr mutation	3/81 (4%)	1/29 (3%)	0/42 (0%)	0/1 (0%)

k13=kelch13.

Parasite positivity at day 3, PCT_1/2_, and *k13* mutations are shown for both treatment groups in table 4. Eight (9%) of the 87 patients treated with artemether–lumefantrine and ten (12%) of the 86 patients treated with artesunate–amodiaquine had parasites with the *k13* Arg561His mutation on day 0 and persistent parasitaemia on day 3. The positivity rate on day 3 was significantly higher in patients harbouring parasites with the *k13* Arg561His mutation than in patients with wild-type parasites in both the artemether–lumefantrine (Fisher's exact test, p=0·0004) and the artesunate–amodiaquine (Fisher's exact test, p=0·0002) groups. Both treatment groups had patients who carried parasites with Arg561His mutations but had no detectable parasites on day 3 (table 4); additionally, both groups contained patients who had detectable parasites on day 3 despite harbouring Arg561 wild-type parasites. The median PCT_1/2_ was similar in patients treated with artemether–lumefantrine (4·21 h) and those treated with artesunate–amodiaquine (4·10 hrs; p=0·75), but 28 (32%) of the 88 patients in the artemether–lumefantrine group and 35 (40%) of the 88 patients in the artesunate–amodiaquine group had a PCT_1/2_ of 5 h or more. The median PCT_1/2_ was significantly higher in patients carrying mutated parasites than in those infected with wild-type parasites, in both the artemether–lumefantrine group (6·1 h *vs* 3·9 h; p<0·0001) and the artesunate–amodiaquine group (6·0 h *vs* 3·6 h; p<0·0001). In the artemether–lumefantrine group, 18 (86%) of 21 patients infected with parasites carrying the Arg561His mutation had a PCT_1/2_ of at least 5 h; the remaining three (14%) patients had a PCT_1/2_ of less than 5 h. Of those treated with artesunate–amodiaquine, 16 (89%) of 18 patients carried parasites with the Arg561His mutation and had a PCT_1/2_ of at least 5 h, whereas only two (11%) patients were infected with mutant parasites but had a PCT_1/2_ of less than 5 h (p<0·0001).

**Table 4:** Parasite clearance half-life and presence or absence of parasites on day 3 among patients with *k13* wild type or Arg561His mutation

	**Parasite clearance half-life**			**Parasites on day 3**	
	Median (IQR), h	≥5 h	<5 h	Positive	Negative
Artemether–lumefantrine (n=87)*					
*k13* wild type on day 0 (n=66)	3·9 (2.81–4·64)	10 (15%)	56 (85%)	3 (5%)	63 (95%)
*k13* with Arg561His mutation on day 0 (n=21)	6·1 (5·26–6·62)	18 (86%)	3 (14%)	8 (38%)	13 (62%)
Artesunate–amodiaquine (n=86)*					
*k13* wild type on day 0 (n=68)	3·6 (2·91–5·10)	18 (26%)	50 (74%)	8 (12%)	60 (88%)
*k13* with Arg561His mutation on day 0 (n=18)	6·0 (5·55–6·37)	16 (89%)	2 (11%)	10 (56%)	8 (44%)

Data are n (%) unless otherwise stated. *k13*=*kelch13*.

* *k13* sequencing was unsuccessful for one sample from the 88 patients in the artemether–lumefantrine group and two samples from the 88 patients in the artesunate–amodiaquine group.

**Figure 2:**
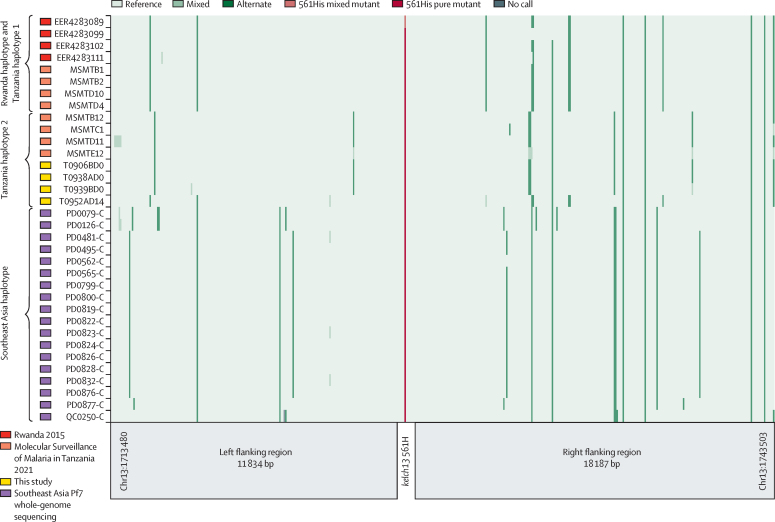
Extended flanking haplotype plot around *k13* among homozygous Arg561His mutants Samples from the current therapeutic efficacy study share the haplotypes previously described in the 2021 survey in Tanzania (Tanzania haplotype 1 and Tanzania haplotype 2). One sample (ERR4283089) has mixed genotypes with both mutant and wild-type alleles. *k13*=*kelch13*.

Haplotype analysis showed that parasites with the *k13* Arg561His mutation in this study were not similar to those with this mutation from southeast Asia and Rwanda, but had the same haplotype (Tanzania haplotype 2) as reported in the parasites sampled in the Kagera region of northwestern Tanzania in the 2021 study (figure 2; appendix p 17). The principal components analysis showed clear evidence of geographical clustering of the parasite from southeast Asia without any mixing with those from Tanzania (appendix p 18). Relatedness network analysis, using identity-by-descent to discern clusters of infections sharing more than 90% identity, showed that highly related parasite pairs were found within Tanzania and within southeast Asia, with no evidence of mixing (appendix p 18), suggesting that Arg561His haplotypes in this study did not originate from southeast Asia.

## Discussion

We show that both artemether–lumefantrine and artesunate–amodiaquine are highly efficacious for the treatment of uncomplicated malaria in children aged 6–120 months in northwestern Tanzania, with respective PCR-corrected ACPRs of 98% and 100%. The *k13* Arg561His mutation was detected in more than 20% of patients at enrolment, a prevalence similar to that reported in the 2021 nationwide survey.^17^ Parasites with the *k13* Arg561His mutation were significantly associated with parasitaemia on day 3. The proportions of patients harbouring parasites with the Arg561His mutation on day 0 and with persistent parasitaemia on day 3 (9% of those treated with artemether–lumefantrine and 12% of those treated with artesunate–amodiaquine) were higher than the WHO threshold of 5%. These findings confirm, for the first time to our knowledge, the presence of ART-R in Tanzania—in the region bordering both Rwanda and Uganda, where ART-R has previously been confirmed. In both the artemether–lumefantrine and the artesunate–amodiaquine groups, the *k13* Arg561His mutation on day 0 was also associated with PCT_1/2_ of 5 h or more—another criterion for confirming ART-R. The risk of day 3 parasitaemia is influenced by initial parasite density, and both day 3 parasitaemia and parasite clearance rates can be affected by the extent of immunity within the population.^26^ Considering that the current study was conducted in a high-transmission region, some clinical implications of the presence of the *k13* Arg561His mutation could possibly have been mitigated by immunity within the study groups. Interindividual differences in the pharmacokinetics of antimalarials can also influence parasite clearance; however, this could not be ascertained in the current study. These factors could potentially account for the 2% of patients treated with artemether–lumefantrine and 9% of patients treated with artesunate–amodiaquine who harboured parasites containing the *k13* Arg561His mutation on day 0 but had no parasitaemia on day 3.

The continued high efficacy of the tested ACTs, even in the presence of ART-R, aligns with previous reports.^3^, ^11^, ^12^, ^13^ Such efficacy could potentially be due to the high efficacy of the partner drugs to the artemisinin derivatives. However, ART-R exposes a greater number of parasites to the partner drug alone, putting these drugs at greater risk of a reduction in efficacy—ART-R has been found to facilitate the spread of partner drug resistance in a previous study.^27^ The higher reinfection rate in patients treated with artemether–lumefantrine than in those treated with artesunate–amodiaquine could be attributed to shorter post-treatment protection from lumefantrine than from amodiaquine and its active metabolite. Such high rates of recurrent infections within 4 weeks of first-line treatment (with artemether–lumefantrine), as observed in this study and in previous studies in the country,^15^, ^16^ have negative health and economic consequences for the population and the health system. Efforts should therefore be directed towards mitigating such breakthrough infections by intensifying control measures to reduce the risk of malaria infections in the population. Artesunate–amodiaquine, the recommended alternative ACT in the country, showed better protective efficacy against new infections in this study and could be considered for use instead of artemether–lumefantrine.

The parasites with the mutations reported in this study were not imported from southeast Asia and were not similar to those from Rwanda; however, the haplotype—Tanzania haplotype 2—was similar to that of parasites previously sampled in the 2021 study in the Kagera region of Tanzania.^17^ The results of the haplotype, principal components analysis, and identity-by-descent analysis suggest that the haplotypes in the samples from the current study were not imported from southeast Asia, and do not provide evidence of the parasites with Arg561His mutations from Rwanda. These results instead indicate that the Tanzania haplotype 2, first detected in 2021,^17^ is spreading in the region. The nature of the parasites detected in the Karagwe district of this region in 2021 and 2022, and the rapid genomic changes within this timeframe, warrant more studies to further understand the evolution of the *k13* Arg561His-mutated parasites and their spread in the region and in neighbouring countries.

In addition to the confirmed ART-R in Eritrea, Rwanda, Tanzania, and Uganda, emergence and spread of the validated *k13* Arg622Ile mutation has also been reported in Ethiopia and Sudan.^28^, ^29^ Furthermore, reports indicate increasing prevalence and spread of validated *k13* mutations in Rwanda and Uganda.^30^, ^31^, ^32^ The emergence and spread of ART-R in Africa is of great concern as the continent accounts for more than 95% of the global malaria burden. The strategy developed by WHO to respond to antimalarial drug resistance in Africa calls on countries to develop a context-specific strategy to respond to this new threat.^7^ Strengthening the monitoring of ACT efficacy and known markers of resistance to artemisinin and partner drugs is one of the pillars of the strategy, and should be among high priority areas for future studies. Lessons learned from intensified nationwide malaria molecular surveillance over the past 5–10 years should be scaled up and used to identify hotspots for *k13* mutations and guide the need for additional therapeutic efficacy studies. Notably, the Kagera region borders both Rwanda and Uganda, where ART-R has also been confirmed, and these areas have high rates of human activity and movement.^18^ Cross-border collaboration between these countries—and others in the Great Lakes region of Africa—in developing harmonised response strategies would strengthen the overall response to the threat of artemisinin and ACT resistance.

To correct treatment outcomes, we used *msp1, msp2*, and *glurp*, instead of following the genotyping method using *msp1, msp2*, and a microsatellite (poly-α, Pfpk2, or TA1) as recommended by WHO for use in a high-transmission setting.^33^ Using *glurp* instead of a microsatellite is more likely to overestimate the cure rate. However, we could not use the newer methods because the protocol had not been optimised in our laboratory, and we wanted to avoid delays in analysing and disseminating the data owing to their potential policy implications. Our laboratory has used the current method for more than 15 years to generate high-quality data in our previous studies. Following the older WHO protocol is unlikely to have affected the conclusions of this study, as the PCR-corrected cure rate was very high. In areas with high transmission, such as Karagwe, the parasites might be cleared more quickly than in lower-transmission areas owing to high levels of immunity within the population. Therefore, the 5-h threshold—which is based on data mainly from southeast Asia—may not be sensitive enough, emphasising the need to reconsider the threshold on the basis of data from high-transmission settings.

This therapeutic efficacy study confirms the presence of ART-R in Tanzania and calls for the development of a context-specific response strategy. The high breakthrough rate of malaria infections after treatment with artemether–lumefantrine should prompt enhanced malaria-control measures to reduce the risk of malaria infections in the population and a discussion regarding the benefits of shifting to an ACT with longer post-treatment protection. Although artemether–lumefantrine and artesunate–amodiaquine showed high efficacy, strengthened surveillance to monitor for decreased efficacy of these combinations, the potential emergence of lumefantrine resistance, and the detection of ART-R in other parts of Tanzania is crucial.

### Contributors

### Data sharing

All sequencing data from the current study are available under accession numbers SAMN41656487–SAMN41656575 at the Sequence Read Archive (http://www.ncbi.nlm.nih.gov/sra); the associated BioProject (https://www.ncbi.nlm.nih.gov/bioproject/) accession number is PRJNA1119461. Clinical and parasitological data have been shared with WHO and will be made freely available after publication by depositing them in WHO public databases. The data include de-identified patient clinical data, a data dictionary, and selective whole genome amplification data.

## Declaration of interests

We declare no competing interests.
