# Supplementary material for: Evidence of artemisinin partial resistance in northwestern Tanzania: clinical and molecular markers of resistance
Source: Lancet Infect Dis. 2024 Nov;24(11):1225–33. doi: 10.1016/S1473-3099(24)00362-1 (PMC11511676; doi:10.1016/S1473-3099(24)00362-1)
Supplement: Supplementary appendix [file mmc1.pdf]

THE LANCET
Infectious Diseases

Supplementary appendix

This appendix formed part of the original submission and has been peer reviewed. We post it as supplied by the authors.

Supplement to: Ishengoma DS, Mandara CI, Bakari C, et al. Evidence of artemisinin partial resistance in northwestern Tanzania: clinical and molecular markers of resistance. *Lancet Infect Dis* 2024; published online Aug 16. https://doi.org/10.1016/S1473-3099(24)00362-1.

SUPPLEMENTARY APPEDIX

Evidence of artemisinin partial resistance in North-western Tanzania: clinical and molecular markers of resistance

Table of contents

Supplementary information on the methods and results 2

References 8

Supplementary table S1: Adverse events reported among patients treated with artemether-lumefantrine and artesunate-amodiaquine 11

Supplementary Table S2: Artemether-lumefantrine genotyping results 12

Supplementary Table S3: Artesunate-amodiaquine genotyping results 14

Supplementary Figure S1 15

Supplementary Figure S2. 16

Supplementary Figure S3. 17

Supplementary Figure S4. 18

Legend to the supplementary figures S1-S4 19

Supplementary information on the methods and results

Study site

This therapeutic efficacy study enrolled children who met the study inclusion criteria and were treated with either artemether-lumefantrine or artesunate-amodiaquine under direct supervision of the study nurse. Enrolled patients were followed up for 28 days through both scheduled and unscheduled visits according to the WHO standard protocol.[1] The study was conducted at Bukangara dispensary in Karagwe district, Kagera region, northwestern Tanzania. The site was selected among the health facilities in Kagera region that were covered in the 2021 nationwide survey done by the project on molecular surveillance of malaria in Tanzania (MSMT). The 2021 MSMT survey reported the prevalence of *k13* Arg561His mutation in Kagera region at 7.7%, but the mutant parasites were only reported in three districts of Karagwe (22.8%), Kyerwa (14.4%) and Ngara (1.4%).[2] Bukangara dispensary was the only facility in Karagwe that was covered by the 2021 survey, and it was therefore selected for this study. Karagwe district, with a population of 385,744 according to the Tanzanian 2022 census, borders Uganda to the north and Rwanda to the west. It also borders Kyerwa district to the north, the districts of Missenyi and Muleba to the east, and Ngara and Biharamulo districts to the south (see Figure 1 in the manuscript). The region has high malaria transmission intensities due to high rainfall, with two peaks after the long (March - June) and short rainy season (December – January). The region has large bodies of water which provide permanent breeding sites and supports malaria transmission throughout the year. The proximity of the study site in Karagwe to Rwanda and Uganda where artemisinin partial resistance (ART-R) has been confirmed, made it suitable for this study. The current study was prompted by the reports of ART-R and was conducted as recommended by WHO in response to results of molecular markers surveillance. It aimed at testing the efficacy of artemether-lumefantrine and artesunate-amodiaquine which are key ACTs in Tanzania, and confirming the presence of ART-R in Kagera region.

Procedures

Screening for eligibility and enrolment were done according to the WHO protocol for TES.[1] Enrolled children had the following inclusion criteria: age between six months and 10 years, axillary temperature ≥37.5^{0}C and reported history of fever in the last 24 h, or with either axillary temperature ≥37.5^{0}C alone or together with history of fever in the

last 24h, confirmed *Plasmodium falciparum* mono-infection with an asexual parasitaemia of 500 - 200,000/μl of blood detected by microscopy, haemoglobin levels greater or equal to 8 g/dL, and able and willing to attend scheduled follow-up visits. Exclusion criteria included general danger signs or symptoms of severe falciparum malaria as defined by WHO, mixed or mono infections with non-falciparum species, severe malnutrition (for children with 6 - 60 months old), co-infections with febrile conditions, anaemia (defined as haemoglobin levels below 8 g/dL), and history of drug reactions. In this study, severe malnutrition was defined as a child who had a mid-upper arm circumference < 110mm for those aged ≤ 59 months or with body mass index of <16 in children aged 6 years and above. Patients who failed to meet the eligibility criteria for enrolment in the study were excluded and treated according to the national treatment guidelines[3].

Patients were enrolled sequentially starting with artemether-lumefantrine and later amodiaquine-artesunate. Children in the artemether-lumefantrine group received a dose twice daily for three consecutive days based on the recommended weight bands: one tablet (20mg artemether and 120mg of lumefantrine) for children with 5 - 14 Kg body weight, two tablets for 15 - 24 Kg body weight, three tablets for 25 - 34 Kg body weight and four tablets for ≥35 Kg body weight. Although all patients were provided with meals during their stay at the study health facility, no food was given at the time of drug administration. The amodiaquine-artesunate group received a daily dose for three days based on the following weight bands: one tablet of artesunate (25mg) and amodiaquine (67.5mg) for children with 4.5 to <9 Kg body weight, one tablet of artesunate (50mg) and amodiaquine (135mg) for 9 to <18 Kg body weight, one tablet of artesunate (100mg) and amodiaquine (270mg) for 18 to <36 Kg body weight. WHO-prequalified artemether-lumefantrine (Cipla Ltd, Mumbai, India) and amodiaquine-artesunate (Winthrop®, Sanofi Aventis, Casablanca, Morocco) were provided by WHO headquarters. The study children were retained at the dispensary for three days. All doses were administered orally and timely under direct observation of experienced nurses and the patients were observed for 30 minutes to ensure no vomiting occurred. If vomiting occurred within 30 minutes, another full dose was administered. Any patient who persistently vomited the study drugs was withdrawn and treated with injectable artesunate followed by a full dose of artemether-lumefantrine according to the national treatment guidelines[3].

Clinical and parasitological assessments were performed at scheduled visits (on days 1, 2, 3, 7, 14, 21, and 28). In addition, parents or guardians were informed that they could bring their children back to the clinic at any time (unscheduled visits) if the child felt unwell. Scheduled follow-up visits were arranged on specified dates, but with an allowance of one day before or after the scheduled day. Parents were reminded by an experienced project staff about the follow-up visits' schedule of their children and this was done at least two 2 days before their visits. For all children who did not come for their scheduled visits on time (10 am), their parents were contacted to know the reasons and follow up was initiated. Those who could not be found on a particular day were then traced and attended on the following day.

Patients with recurrent infections in artemether-lumefantrine group were treated with amodiaquine-artesunate while those in amodiaquine-artesunate group were treated with artemether-lumefantrine because both drugs are recommended by the Tanzanian National Malaria Control Programme (NMCP) for the treatment of uncomplicated malaria. Artemether-lumefantrine is the first line antimalarial drug in Tanzania while amodiaquine-artesunate is the alternative ACT which is used when artemether-lumefantrine is contraindicated, not recommended, or unavailable. The safety of study drugs was assessed at each follow-up visit or during unscheduled visits by asking parents or guardians about the occurrence and nature of adverse events, and serious adverse events. Thereafter, clinical assessment was done by experienced study clinicians, and if required, laboratory investigations were also performed. The findings were recorded in appropriate case report forms and defined, as well as classified according to the WHO protocol.[1] In case a serious adverse event was observed, the protocol directed that it should be reported within 24 hours to the principal investigator who would report the event to the National Institute for Medical Research (NIMR), WHO, and the Medical Research Coordinating Committee of NIMR (NIMR-MRCC). No serious adverse events were reported in this study, but all patients with adverse events were thoroughly investigated and managed according to the WHO protocol.[1]

Thick and thin blood smears were taken through finger prick by an experienced laboratory technician and dried in a safe place in the laboratory which was set up at the dispensary for this TES. Thin smears were fixed with methanol and all smears were

stained with Giemsa (5%) for 45 minutes. The smears were examined to detect the presence of *Plasmodium falciparum* and to estimate parasite density before treatment (day 0) and at each scheduled (days 1, 3, 7, 14, 21 and 28) or unscheduled visit according to WHO protocol.[1] In addition, blood smears were taken every 8 h until two consecutive smears were negative or the patient reached day 3 before clearing the parasites, to assess parasite clearance half-life ($PCT_{1/2}$). To ensure high quality microscopy data were generated, all blood slides were read by two independent expert microscopists and the two parasite densities were averaged. Initial reading was done at the dispensary and the results were used for patients' assessment during the 28 – days of follow-up. The second reading was done in the laboratory at NIMR after completing the study. If there were discrepancies in parasite positivity, parasite species or difference in parasite density of more than 50% between the two readings, the slide was re-examined by a third independent microscopist. After the third reading, the two closest results were taken and used for analysis. Any additional discrepancy was resolved by the three experts who discussed the results and agreed on the reading. A slide was declared negative if no parasites were seen after counting 1000 leukocytes. The presence of gametocytes at inclusion in the study or at follow-up visits was also recorded, but was not deemed as an exclusion criterion. In addition, 100 fields of the thick smears were examined on day 0 to rule out mixed infections. In cases of doubt, the thin film was examined for confirmation. Before launching of the study, all study staff were trained on the study protocol, all source documents, and other study materials. They were also trained on good clinical practices and good clinical laboratory practices. During the conduct of the study, supervision and monitoring visits were conducted by experienced investigators and data were validated against source documents to ensure that the study complied with the WHO standard protocol.[1] Supportive supervisions also ensured that clinical and parasitological assessment procedures and the quality control procedures for parasite count were followed.

Genotyping and Molecular analysis

Blood samples were collected as dried blood spots (DBS) on Whatman #3 filter paper (GE Healthcare Life Sciences, PA, USA), from each patient on day 0 and on the day of parasite recurrence (from day 7 onwards). They were dried, stored in individual plastic bags with desiccant until analysis, protecting them from light, moisture, and extreme temperatures.

Parasite DNA was extracted from DBS and used for PCR-based analysis to differentiate reinfection from recrudescence, as well as sequencing of key mutations. Parasite DNA was extracted using QIAamp DNA blood mid-kit (Qiagen GmbH, Hilden, Germany), according to the manufacturer's instructions. The extraction was done from three 3 mm punches of each DBS sample, and these were determined to generate sufficient DNA and DNA of high quality according to the protocol currently used by the NIMR Genomics Laboratory[4,5]. Paired samples (day 0 and day of parasite recurrence) were genotyped using merozoite surface proteins 1 and 2 (*msp1* and *msp2*), and glutamate rich protein (*glurp*) genes to distinguish recrudescent from new infections according to WHO protocol [6].

DNA extracted from samples collected on day 0 (pre-treatment) and on the day of recurrent infections were analysed for mutations in *k13* gene and *Plasmodium falciparum* multidrug resistance 1 (*Pfmdr1)* genes according to the protocols described earlier [7,8]. Whole genome sequencing was done on 49 *k13* Arg561His (mutant) and 34 *k13* Arg561 (wildtype) DNA samples for in-depth analyses including assessment of extended haplotypes around the gene. Selective whole genome amplification was done, followed by library preparation for Illumina sequencing using the Adapterama protocol as previously described[9]. The libraries were processed and sequenced using 2X150bp chemistry on a NovaSeq6000 at the University of North Carolina. We downloaded publicly available whole genome sequence data (n = 25) from *Plasmodium falciparum* isolates carrying Arg561His mutants, collected in 2014/15 in Rwanda[10], Kagera region of northwestern Tanzania in 2021 (n = 9)[2], and Southeast Asia (SEA, n = 42) from Pf7K website.[11] The reads (bam files) from all data obtained above and those generated from this study (n=83) were processed and joint genotyping was performed using GATK4, following previously published methods.[12] VCF files generated from combined data went through additional filtering using VCFtools to retain high quality single nucleotide polymorphisms (SNPs) for downstream haplotype, principal component analysis and identity by descent analysis. The principal component analysis was performed in R using the SNPRelate package version 1.16.1[13]. A high-performance computing toolset was used for relatedness analysis and principal component analysis of single nucleotide polymorphism data.[13] For all pairwise comparisons of parasite samples across four data sets, we estimated identity-by-descent using isoRelate as described previously, which

infers identity-by-descent estimates under a probabilistic model that accounts for recombination[14]. The identity-by-descent analyses are used for measuring population dynamics and selection in recombining pathogens.[14] All figures were plotted and visualised using the ggplot2 R package.

Results

There was no serious adverse events and the reported adverse events were not considered to be related to the treatment with either of the two ACTs. The reported adverse events included cough, runny nose, abdominal pain, fever, nausea and diarrhoea (Table S1). Most of the adverse events occurred in the amodiaquine-artesunate group and majority of the patients in both groups had only one episode of adverse event. Runny nose, abdominal pain, fever, and nausea were more frequent among the amodiaquine-artesunate-treated patients.

The *k13* gene was successfully sequenced in 99% (87/88) of the pre-treatment samples (day 0) in the artemether-lumefantrine group, and 98% (86/88) of the pre-treatment samples in the amodiaquine-artesunate group. In addition, 94% (29/31) of the post-treatment samples with recurrent parasitaemia in artemether-lumefantrine group yielded interpretable results. In the amodiaquine-artesunate group, 100% (2/2) of the post-treatment samples with recurrent parasitaemia had interpretable results (Table 3). All pre-treatment samples (100%) in both groups and all post-treatment samples in the artesunate-amodiaquine group (100%) were successfully analysed for the region 1 of the *Pfmdr1* gene. In the artemether-lumefantrine group, 94% (29/31) of the post-treatment group were successfully analysed. For the region 2 of the *Pfmdr1* gene, 92% (81/88) of the pre-treatment samples and 94% (29/31) of the post-treatment samples in the artemether-lumefantrine group yielded interpretable results. In the amodiaquine-artesunate group, only 48% (42/88) of the pre-treatment samples and 50% (1/2) of post-treatment samples yielded interpretable results.

Mutations in the *Pfmdr1* gene (codons 86, 184 and 1246) suspected to be associated with reduced sensitivity to 4-aminoquinolines, including lumefantrine were analysed based on single nucleotide polymorphisms at the three codons and haplotypes. The analysis covered the three single nucleotide polymorphisms (Asn86Tyr, Tyr184Phe and

Asp1246Tyr) and their corresponding haplotypes associated with reduced susceptibility to lumefantrine.[15,16] In both pre-treatment and recurrent infections, the Asp86 wildtype was fixed (100%) in the artemether-lumefantrine group, and in the amodiaquine-artesunate group. The mutation at Asp1246Tyr mutation was reported in 3/81 (4%) pre-treatment samples, and 1/29 (3.5%) post-treatment samples in the artemether-lumefantrine group. The mutation was not detected in the amodiaquine-artesunate group (all samples had the wildtype allele). In contrast, the Tyr184Phe mutation was reported in 37/88 (42%) pre-treatment samples, and 12/29(41%) in the post-treatment samples in artemether-lumefantrine. The Tyr184Phe mutation was also reported in 37/88 (42%) pre-treatment samples in the amodiaquine-artesunate group. In the artemether-lumefantrine group, the Asn-Phe-Asp haplotype was reported in 33/81 (41%) pre-treatment samples while the Asn-Tyr- Asp haplotype was reported in 45/81 (56%) pre-treatment samples. In the post-treatment samples, Asn-Phe-Asp haplotype was reported in 12/28(43%) samples and Asn-Tyr- Asp haplotype occurred in 15/28 (54%) samples. The Asn-Phe-Asp haplotype was detected in 16/42 (38.1%) pre-treatment samples and 26/42 (61.9%) post-treatment samples among amodiaquine-artesunate treated-patients.

Another haplotype which was only detected in the artemether-lumefantrine group was Asn-Tyr-Tyr and it occurred in 3/81 (4%) pre-treatment samples, and 1/28 (4%) post-treatment sample. The analysis was done for the polymorphisms and haplotypes in the artemether-lumefantrine group only and showed that there was no significant association with recurrent infections or recrudescent for any of the single nucleotide polymorphism or haplotypes ($p>0.41$). These findings suggest that none of the single nucleotide polymorphism or haplotype were selected by artemether-lumefantrine treatment. The analysis could not be done for amodiaquine-artesunate because there were only two patients with recurrent infections.

References

1. WHO. Tools for monitoring antimalarial drug efficacy. https://www.who.int/teams/global-malaria-programme/case-management/drug-efficacy-and-resistance/tools-for-monitoring-antimalarial-drug-efficacy (Accessed 03 May 2024).
2. Juliano, J. J. *et al.* Country wide surveillance reveals prevalent artemisinin partial

resistance mutations with evidence for multiple origins and expansion of high level sulfadoxine-pyrimethamine resistance mutations in northwest Tanzania. *medRxiv* (2023) doi:10.1101/2023.11.07.23298207.

3. MoH. National Guidelines for Malaria Diagnosis, Treatment and Preventive Therapies 2020. 2021. Dodoma, Ministry of Health. (2020).
4. Popkin Hall, Z. R. *et al.* Malaria species positivity rates among symptomatic individuals across regions of differing transmission intensities in Mainland Tanzania. *J. Infect. Dis.* (2023) doi:10.1093/infdis/jiad522.
5. Popkin Hall, Z. R. *et al.*Prevalence of non-falciparum malaria infections among asymptomatic individuals in four regions of Mainland Tanzania. Parasites and Vectors; March 2024; https://parasitesandvectors.biomedcentral.com/articles/10.1186/s13071-024-06242-4.
6. WHO. Methods for surveillance of antimalarial drug efficacy methods for surveillance of antimalarial drug efficacy. https://apps.who.int/iris/bitstream/handle/10665/44048/9789241597531_eng.pdf (Accessed 03 May 2024)..
7. Ishengoma, D. S. *et al.* Efficacy and safety of artemether-lumefantrine for the treatment of uncomplicated malaria and prevalence of Pfk13 and Pfmdr1 polymorphisms after a decade of using artemisinin-based combination therapy in mainland Tanzania. *Malar. J.* **18**, 88 (2019).
8. Ariey, F. *et al.* A molecular marker of artemisinin-resistant Plasmodium falciparum malaria. *Nature* **505**, (2014).
9. Oyola, S. O. *et al.* Whole genome sequencing of Plasmodium falciparum from dried blood spots using selective whole genome amplification. *Malar. J.* **15**, 1–12 (2016).
10. Uwimana, A. *et al.* Emergence and clonal expansion of in vitro artemisinin-resistant Plasmodium falciparum kelch13 R561H mutant parasites in Rwanda. *Nat. Med.* **26**, (2020).
11. MalariaGEN *et al.* Pf7: an open dataset of genome variation in 20,000 worldwide samples. *Wellcome Open Res* **8**, 22 (2023).
12. Optimized_GATK4_pipeline: Part1: fastq and bam processing and quality check. Part2: variant calling, filtering and annotation. Github https://github.com/Karaniare/Optimized_GATK4_pipeline.

13. Zheng, X. *et al.* A high-performance computing toolset for relatedness and principal component analysis of SNP data. *Bioinformatics* **28**, (2012).
14. Henden, L., Lee, S., Mueller, I., Barry, A. & Bahlo, M. Identity-by-descent analyses for measuring population dynamics and selection in recombining pathogens. *PLoS Genet.* **14**, (2018).
15. Venkatesan, M. *et al.* Polymorphisms in Plasmodium falciparum chloroquine resistance transporter and multidrug resistance 1 genes: parasite risk factors that affect treatment outcomes for P. falciparum malaria after artemether-lumefantrine and artesunate-amodiaquine. *Am. J. Trop. Med. Hyg.* **91**, (2014).
16. Malmberg, M. *et al.* Plasmodium falciparum drug resistance phenotype as assessed by patient antimalarial drug levels and its association with pfmdr1 polymorphisms. *J. Infect. Dis.* **207**, (2013).

Supplementary table S1: Adverse events reported among patients treated with artemether-lumefantrine and amodiaquine-artesunate

Adverse event	AL (n=88)			ASAQ (n=88)		
	AES episodes/person			AES episodes/person		
	Total	One	Two	Total	One	Two
Cough	29 (33)	26 (29.5)	3 (3.4)	29 (33)	28 (31.8)	1 (1.1)
Runny nose	3 (3.4)	3 (3.4)	0 (0)	13 (14.8)	11 (12.5)	2 (2.3)
Abdominal pain	2 (2.3)	2 (2.3)	0 (0)	11 (12.5)	9 (10.2)	2 (2.3)
Fever	3 (3.4)	3 (3.4)	0 (0)	10 (11.4)	9 (10.2)	1 (1.1)
Nausea	1 (1.1)	1 (1.1)	0 (0)	3 (3.4)	3 (3.4)	0 (0)
Loose stool	0 (0)	0 (0)	0 (0)	2 (2.3)	2 (2.3)	0 (0)
Loss appetite	1 (1.1)	1 (1.1)	0 (0)	1 (1.1)	1 (1.1)	0 (0)
Watery diarrhoea	1 (1.1)	1 (1.1)	0 (0)	1 (1.1)	1 (1.1)	0 (0)
Bee sting on the face	1 (1.1)	1 (1.1)	0 (0)	0 (0)	0 (0)	0 (0)
Slight Pale	0 (0)	0 (0)	0 (0)	1 (1.1)	1 (1.1)	0 (0)
vomiting	1 (1.1)	1 (1.1)	0 (0)	0 (0)	0 (0)	0 (0)
Urination	**0 (0)**	**0 (0)**	**0 (0)**	**1 (1.1)**	**1 (1.1)**	**0 (0)**
**Total**	**42 (47.7)**	**39 (44.3)**	**3 (3.4)**	**72 (81.8)**	**66 (75)**	**6 (6.8)**

AL = artemether-lumefantrine, ASAQ = artesunate-amodiaquine, n = number of patients, % = percentage , *number of events per patient to present number of patients who experienced a single or multiple episode of AEs.

Supplementary Table S2: Artemether-lumefantrine genotyping results

SAMPLE ID	*MSP1*				*MSP2*			*GLURP*		
	K1	***Mad20***	***RO33***	***Results; msp1***	***IC1***	***FC27***	***Results ; msp2***		***Results ;glurp***	***Results; all markers***
T0905A D0	254	200	0	R	457	487	NI	929	NI	NI
T0905A D28	246	0	0		369	0		1023		
T0906A D0	225	0	0	R	498	638	R	1177	R	R
T0906A D21	220	0	113		502	0		1146		
T0908A D0	217	232	120	R	453	656	NI	994	NI	NI
T0908A D28	0	0	122		561	0		1175		
T0912A D0	231	236	0	NI	480	625	NI	1302	NI	NI
T0912A D21	257	0	0		441	0		1215		
T0914A D0	244	0	116	NI	473	673	NI	1181	NI	NI
T0914A D21	0	194	0		629	0		1285		
T0915A D0	271	0	126	NI	449	557	NI	1181	R	NI
T0915A D21	205	0	0		0	646		1194		
T0918A D0	258	0	128	NI	0	714	NI	1190	NI	NI
T0918A D21	239	0	0		0	643		1371		
T0924A D0	263	228	0	R	515	624	NI	986	NI	NI
T0924A D14	265	0	0		0	674		1445		
T0925A D0	0	0	137	NI	511	0	NI	1213	R	NI
T0925A D21	260	0	0		484	686		1229		
T0926A D0	283	0	0	NI	460	684	NI	1200	NI	NI
T0926A D28	0	241	0		535	0		1272		
T0933A D0	240	0	152	NI	499	608	NI	1230	NI	NI
T0933A D28	271	303	0		723,605	591		1160		
T0934A D0	311	294	0	NI	0	713	NI	1228	NI	NI
T0934A D28	322	0	0		496	0		1169		
T0935A D0	325	0	0	NI	522	602	NI	829	NI	NI
T0935A D28	0	0	165		0	560		1028		
T0936A D0	0	245	166	R	557	624	R	1355	NI	NI
T0936A D28	370	0	166		567	633		1147		
T0937A D0	247	272	0	NI	584	0	NI	1177	NI	NI
T0937A D28	0	0	173		634	611		1091		
T0939A D0	0	261	0	NI	595	0	NI	1373	NI	NI
T0939A D28	0	0	164		652,558	0		1131		

T0944A D0	202	0	0	NI	0	616	NI	1009	R	NI
T0944A D21	251	264	0		431	541		1057		
T0949A D0	228	0	0	R	0	548	NI	1204	R	NI
T0949A D21	222	244	0		0	526		1230		
T0950A D0	0	254	0	NI	0	454	NI	940	NI	NI
T0950A D21	234	0	0		433	0		1033		
T0951A D0	0	213	0	NI	514	415	R	1042	NI	NI
T0951A D28	0	0	131		573,510	0	NI	898	NI	NI
T0952A D0	214	274	301	R	630	566	NI	917	NI	NI
T0952A D14	208	0	0		0	530		1037		
T0953A D0	234	303	123	R	489	0	NI	1035	NI	NI
T0953A D28	225	296	302		666	537		948		
T0958A D0	283	0	131	NI	0	547	NI	1052	R	NI
T0958A D28	209	0	0		632	0		1077		
T0964A D0	237	219	0	R	609	454	NI	987	ND	NI
T0964A D21	0	225	0		661	0		0		
T0966A D0	262	0	0	NI	670	0	NI	1003	R	NI
T0966A D21	238	0	151		611	0		1005		
T0967A D0	245	299	0	ND	518,485	582	ND	990	ND	ND
T0967A D21	0	0	0		0	0		0		
T0968A D0	0	366	171	NI	0	498	R	1017	R	NI
T0968A D14	0	0	159		0	491		964		
T0977A D0	303	0	0	NI	648	589	NI	1053	R	NI
T0977A D21	0	0	169		531	0		1020		
T0981A D0	252	0	0	ND	658	534	ND	1018	ND	ND
T0981A D21	0	0	0		0	0		0		
T0982A D0	244	291	0	R	0	604	NI	992	NI	NI
T0982A D21	0	285	366		605	0		965		
T0984A D0	263	0	0	NI	718	627	NI	922	NI	NI
T0984A D21	290	0	0		643	606		860		

NI = New infection, R = Recrudescent, ND = not determine (non-determined)

MSP2, = merozoite surface protein 1, MSP2 = merozoite surface protein 2 and GLURP = glutamate rich protein.

Supplementary Table S3: Amodiaquine-artesunate genotyping results

SAMPLE ID	***MSP1***				***MSP2***			***GLURP***		
	K1	***Mad20***	***RO33***	***Results; msp1***	***IC1***	***FC27***	***Results; msp2***		***Results;glurp***	***Results; all markers***
T0928B D0	373	0	111	NI	405	437	NI	868	NI	NI
T0928B D14	218	0	0		470	0		641		
T0978B_D0	292	0	0	ND	399	301	NI	941	NI	NI
T0978B_D21	200	0	0		619/565	0		763		

NI = New infection, R = Recrudescent, ND = not determine (non-determined)

MSP2, = merozoite surface protein 1, MSP2 = merozoite surface protein 2 and GLURP = glutamate rich protein.

Supplementary Figure S1

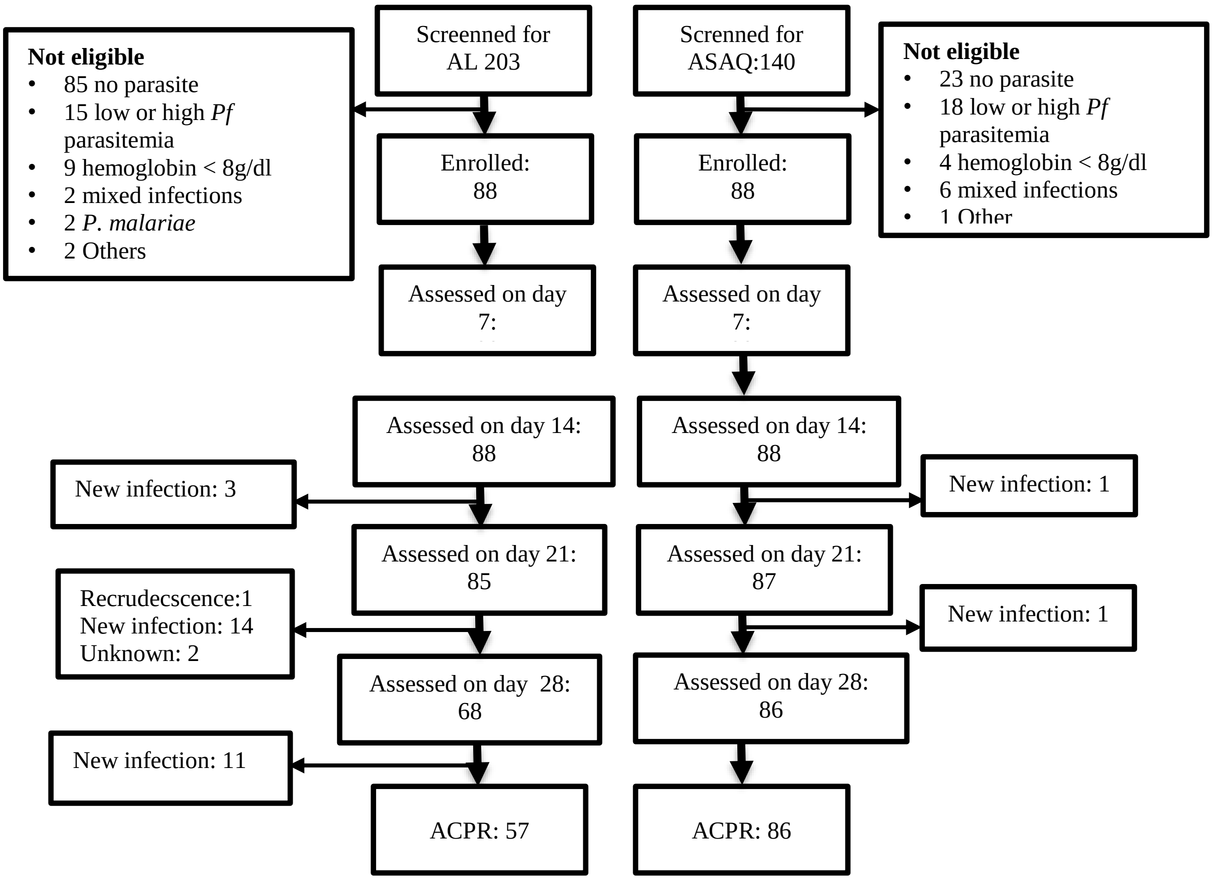

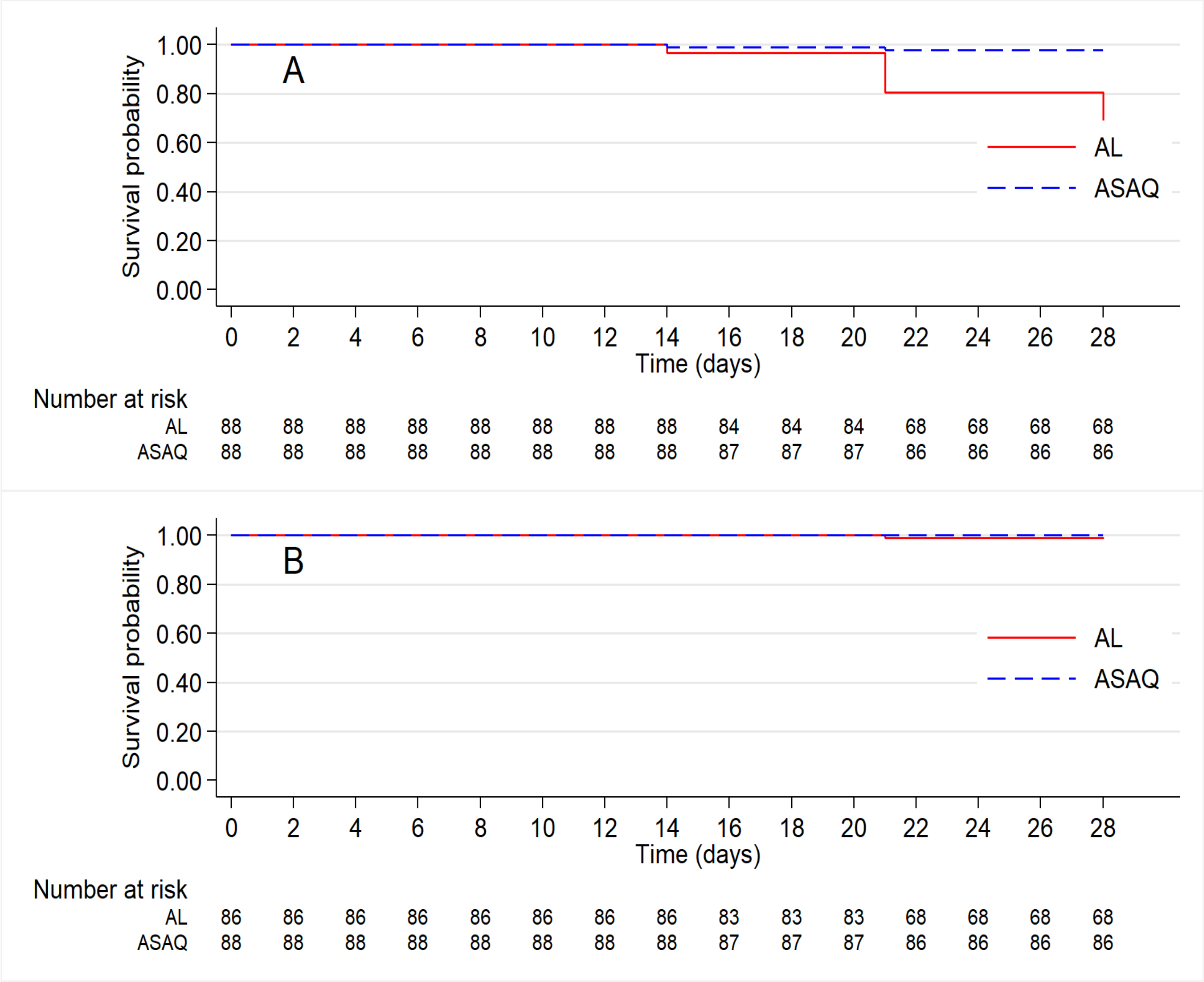
A
B
Survival probability
1.00
0.80
0.60
0.40
0.20
0.00
0 2 4 6 8 10 12 14 16 18 20 22 24 26 28
Time (days)
AL
ASAQ
Number at risk
AL 88 88 88 88 88 88 88 88 84 84 84 68 68 68 68
ASAQ 88 88 88 88 88 88 88 88 87 87 87 86 86 86 86
Number at risk
AL 86 86 86 86 86 86 86 86 83 83 83 68 68 68 68
ASAQ 88 88 88 88 88 88 88 88 87 87 87 86 86 86 86

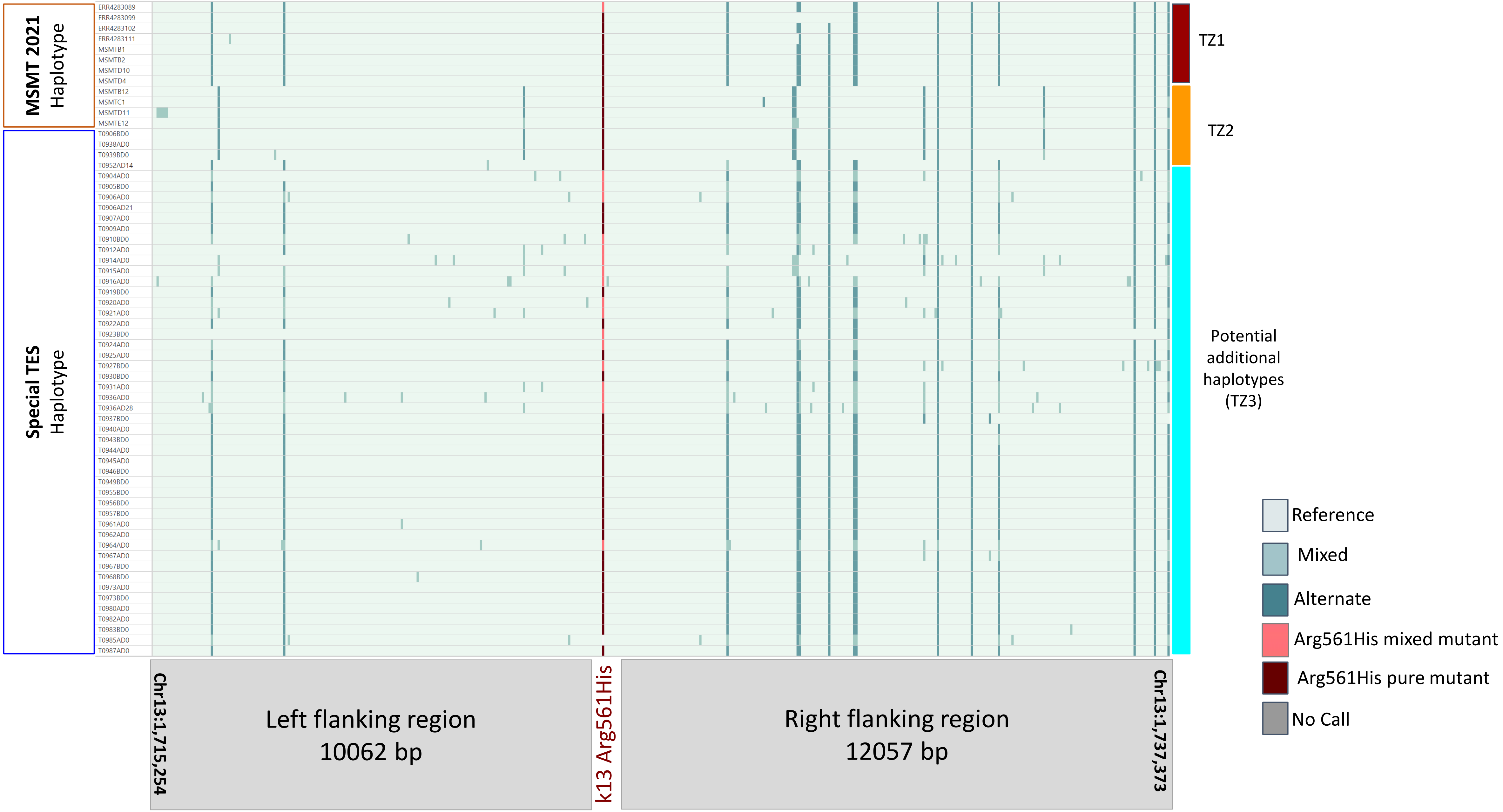
MSMT 2021
Haplotype
Special TES
Haplotype
ERR4283089
ERR4283099
ERR4283102
ERR4283111
MSMTB1
MSMTB2
MSMTD10
MSMTD4
MSMTB12
MSMTC1
MSMTD11
MSMTE12
T0906BD0
T0938AD0
T0939BD0
T0952AD14
T0904AD0
T0905BD0
T0906AD0
T0906AD21
T0907AD0
T0909AD0
T0910BD0
T0912AD0
T0914AD0
T0915AD0
T0916AD0
T0919BD0
T0920AD0
T0921AD0
T0922AD0
T0923BD0
T0924AD0
T0925AD0
T0927BD0
T0930BD0
T0931AD0
T0936AD0
T0936AD28
T0937BD0
T0940AD0
T0943BD0
T0944AD0
T0945AD0
T0946BD0
T0949BD0
T0955BD0
T0956BD0
T0957BD0
T0961AD0
T0962AD0
T0964AD0
T0967AD0
T0967BD0
T0968BD0
T0973AD0
T0973BD0
T0980AD0
T0982AD0
T0983BD0
T0985AD0
T0987AD0
TZ1
TZ2
Potential additional haplotypes (TZ3)
Chr13:1,715,254
Left flanking region
10062 bp
k13 Arg561His
Right flanking region
12057 bp
Chr13:1,737,373
Reference
Mixed
Alternate
Arg561His mixed mutant
Arg561His pure mutant
No Call

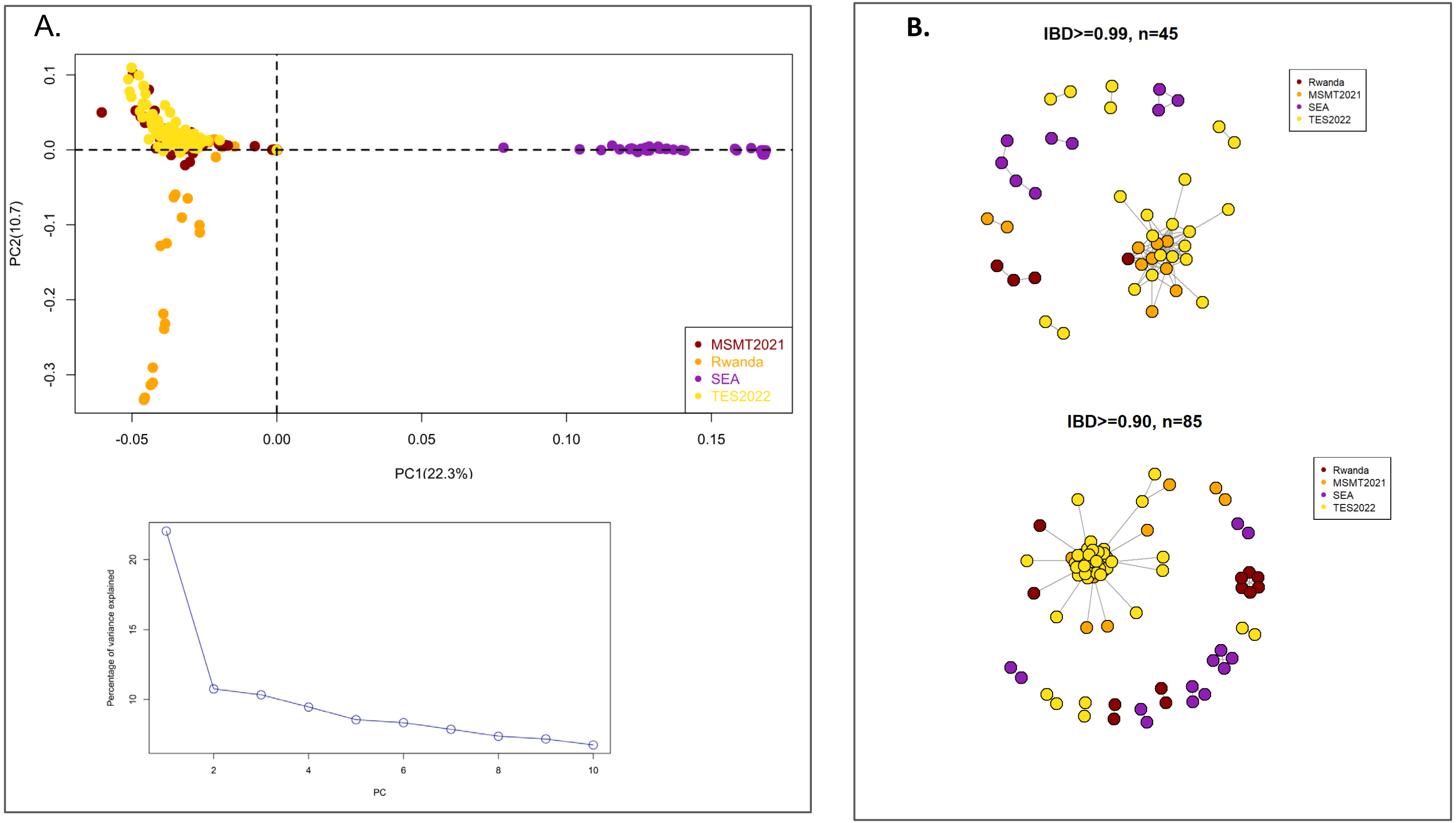
A.
PC2(10.7)
0.1
0.0
-0.1
-0.2
-0.3
-0.05
0.00
0.05
0.10
0.15
PC1(22.3%)
MSMT2021
Rwanda
SEA
TES2022
Percentage of variance explained
20
15
10
2
4
6
8
10
PC
B.
IBD>=0.99, n=45
Rwanda
MSMT2021
SEA
TES2022
IBD>=0.90, n=85
Rwanda
MSMT2021
SEA
TES2022

Legend to the supplementary figures S1-S4

Supplementary Figure S1. Trial profile showing the flow of patients during screening, enrolment and follow-up

Supplementary Figure S2. Kaplan-Meir survival curves of cumulative treatment success for artemether-lumefantrine (red line) and artesunate-amodiaquine (blue dotted line). "A": uncorrected PCR ; "B" corrected PCR.

Supplementary Figure S3. Extended flanking haplotype plot around *k13* among all Arg561His mutants in the current therapeutic efficacy study and from samples previously reported from Kagera Tanzania in 2021. The first haplotype described in Kagera in 2021 [Tanzania haplotype one (TZ1)] is shown in the in dark red on the right. The second Kagera 2021 haplotype [Tanzania haplotype two (TZ2)] and some samples from the current study are shown in orange on the right. The other successfully whole genome sequenced samples from the current are shown in cyan on the right. A highly conserved left flanking haplotype is seen across all samples.

Supplementary Figure S4. Population structure of R56H *P. falciparum* parasite populations from SEA, Tanzania and Rwanda. A) Principal component analysis of Arg561His *Plasmodium falciparum* parasites (top) and percentage of variance explained (bottom). Colours indicate geographic origin and dots indicate individual parasites. B) Relatedness network of Arg56His *Plasmodium falciparum* genome pairs having different proportions of identity-by-descent sharing. Each node identifies a unique sample, and an edge is drawn between two samples if their genomes equal or exceed specified identity-by-descent sharing threshold. Both principal component analysis and identity-by-descent analysis revealed clustering of South-east Asia haplotypes from rest of parasites and confirm our findings that the parasites with Arg561His mutation in Tanzania did not originate in South-east Asia.
